# Gene expression differences during the heterogeneous progression of peripheral atherosclerosis in familial hypercholesterolemic swine

**DOI:** 10.1186/1471-2164-14-443

**Published:** 2013-07-03

**Authors:** Martin Bahls, Christopher A Bidwell, Juan Hu, Armando Tellez, Greg L Kaluza, Juan F Granada, Christian G Krueger, Jess D Reed, M Harold Laughlin, William G Van Alstine, Sean C Newcomer

**Affiliations:** 1 Department of Health and Kinesiology, Purdue University, West Lafayette, IN, USA; 2 Universitätsmedizin Greifswald, Klinik und Poliklinik für Innere Medizin B, Ferdinand-Sauerbruch-Str., Greifswald, Germany; 3 Department of Animal Science, Purdue University, West Lafayette, IN, USA; 4 Department of Statistics, Purdue University, West Lafayette, IN, USA; 5 Skirball Center for Cardiovascular Research, Cardiovascular Research Foundation, New York, USA; 6 Department of Animal Science, University of Wisconsin, Madison, WI, USA; 7 Department of Biomedical Sciences, University of Missouri, Columbia, MO, USA; 8 Department of Veterinary Pathobiology, Purdue University, West Lafayette, IN, USA; 9 California State University San Marcos, San Marcos, CA, USA

**Keywords:** Peripheral arterial disease, Atherosclerosis, Vascular biology, Peripheral vasculature, Gene expression

## Abstract

**Background:**

The heterogeneous progression of atherosclerotic disease in the peripheral arteries is currently not well understood. In humans, artery specific disease progression is partly attributed to the local hemodynamic environments. However, despite similar hemodynamic environments, porcine brachial arteries are protected while femoral arteries are highly susceptible to advanced lesion formation. The aim of this investigation was to determine whether artery specific gene expression patterns contribute to the uneven distribution of peripheral arterial disease (PAD) in Rapacz Familial-Hypercholesterolemic (FHC) swine.

**Results:**

Histological results confirmed rapid atherosclerotic disease progression in femoral but not brachial arteries. A total of 18,922 probe sets had sufficient signal abundance. A main effect for age and artery was observed for 1784 and 1256 probe sets, respectively. A significant age x artery interaction was found for 184 probe sets. Furthermore, comparison between arteries found a decrease from 714 to 370 differentially expressed transcripts from nine months to two years of age. Gene ontology analysis of the 56 genes with a main effect for artery and an age x artery interaction identified vascular smooth muscle contraction as enhanced biological signaling pathway.

**Conclusion:**

This is the first investigation to report that the total number of differential genes decreases with diverging atherosclerotic disease pattern between porcine brachial and femoral arteries.

## Background

Peripheral arterial disease (PAD) affects 8 to 12 million Americans and significantly increases the risk for myocardial infarction and stroke [1]. The heterogeneous progression of atherosclerotic disease in the peripheral arteries is currently not very well understood. Despite the systemic effects of atherogenic risk factors like smoking and a high fat diet [2], not all peripheral conduit arteries are equally susceptible to PAD. Specifically, the brachial artery is known to be protected against advanced lesion formation whereas the femoral artery is highly disease susceptible [3-6]. In humans, this heterogeneous disease distribution between upper and lower limb arteries can partly be attributed to location dependent hemodynamic environments and a blood pressure gradient due to upright posture [3,7-9]. However, in the porcine circulatory system, a human-like disease distribution in the brachial and femoral arteries has been reported despite the absence of this pressure gradient [3-6,10]. In addition, porcine brachial and femoral arteries have similar mean arterial blood pressure, mean blood velocity, and shear rate [10]. Therefore, hemodynamics cannot fully account for the heterogeneous distribution of PAD between brachial and femoral arteries.

Others have hypothesized that the uneven onset and progression of atherosclerosis may be explained by artery specific transcriptomes [2,11]. Artery specific gene expression profiles, possibly a result of differences in angiogenesis and vasculogenesis, have previously been identified and may influence atherosclerotic disease susceptibility [12-14]. The heterogeneous progression of atherosclerosis is exemplified in porcine coronary arteries, which had the fastest rate of lesion development when compared to thoracic and carotid arteries of the same animals [15]. In addition, several studies have investigated gene expression in atherosusceptible arteries to characterize atherosclerotic gene expression patterns. Gene expression differences between arteries with varying atherogenic properties were associated with inflammation and plaque stability [15]. Furthermore, a comparison of gene expression patterns between endothelial cells (EC) of porcine coronary and iliac arteries identified several inflammatory pathways to be up-regulated in the atherosusceptible arteries [16]. Albeit, these investigations provide a description and characterization of atherosclerotic disease stage specific transcriptomes, a mechanism explaining artery specific disease susceptibilities is an important gap in our knowledge. Understanding the progression of atherosclerosis will be critical for developing treatment options, while an appreciation for atheroprotective mechanisms underlying the resistance to advanced atherosclerotic disease in brachial arteries despite being exposed to similar risk factors may provide new preventative therapeutics.

Consequently, the aim of this study was to investigate how artery specific disease progression influences gene expression patterns between atheroprotected brachial and atherosusceptible femoral arteries in Rapacz familial hypercholesterolemic (FHC) swine. In addition to a high similarity of the porcine and human cardiovascular system, Rapacz FHC swine rapidly develop human-like peripheral atherosclerotic disease due to an allelic variant for apolipoprotein B and a low-density lipoprotein receptor dysfunction [17-19]. Despite similar local hemodynamic environments, this breed has atheroprotected brachial and –susceptible femoral arteries [10]. Therefore, the Rapacz FHC swine provides an excellent model to compare gene expression patterns between the progression of atherosclerotic disease in atheroprotected brachial and atherosusceptible femoral arteries. Since we previously identified genes associated with Wnt-signaling, extracellular matrix, and biological adhesion, with different levels of relative transcript abundance between healthy brachial and femoral arteries in 13 day old Rapacz FHC swine [20], we hypothesized that these differences would drive the heterogeneous progression of atherosclerotic disease. Therefore, we expected to see an increase in the total number of genes with differential gene expression between arteries with age and progression of atherosclerotic disease. Furthermore, we suspected that the rapid progression of atherosclerotic disease in the femoral artery would result in an increase in genes previously associated with immune response and inflammation.

## Results

### Histology

**Figure 1:**
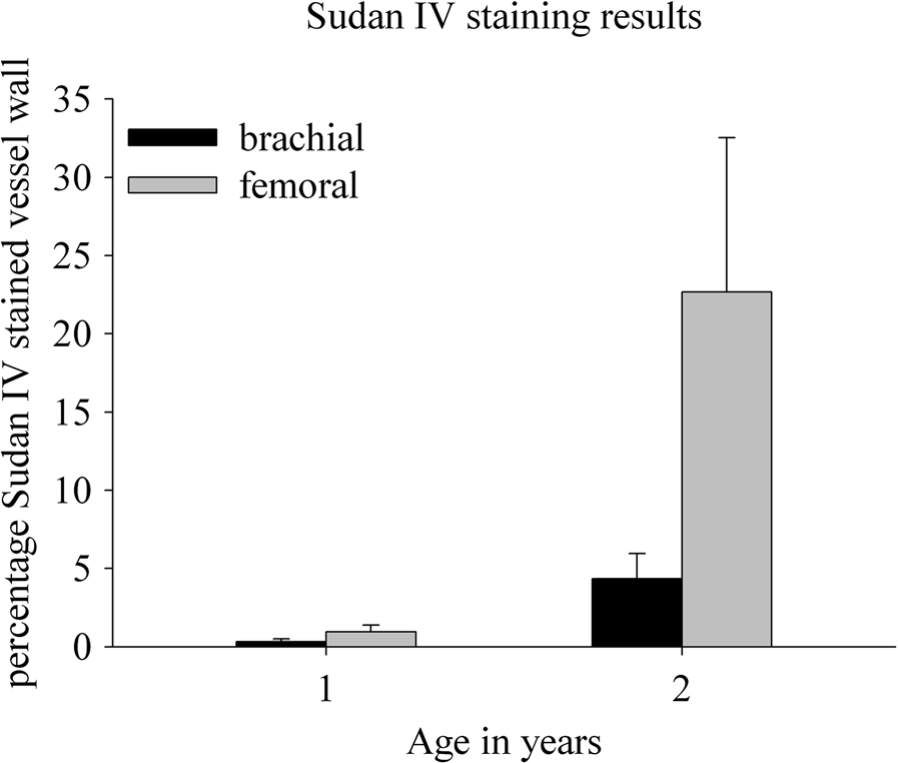
Differential Sudan IV staining between brachial and femoral arteries from 1 and 2 year old Rapacz FHC swine.

In nine month old animals, macroscopic Sudan IV staining showed similar amounts of lipid depositions in the endothelial and sub-endothelial layer of brachial (0.33 ± 0.16%) and femoral (0.93 ±0.43%) artery walls. However, brachial arteries (4.3 ± 1.62%) from two year old animals showed a strong trend (p = 0.09) toward reduced lipid depositions compared to femoral arteries (22.0 ± 9.88%) (Figure 1).

**Figure 2:**
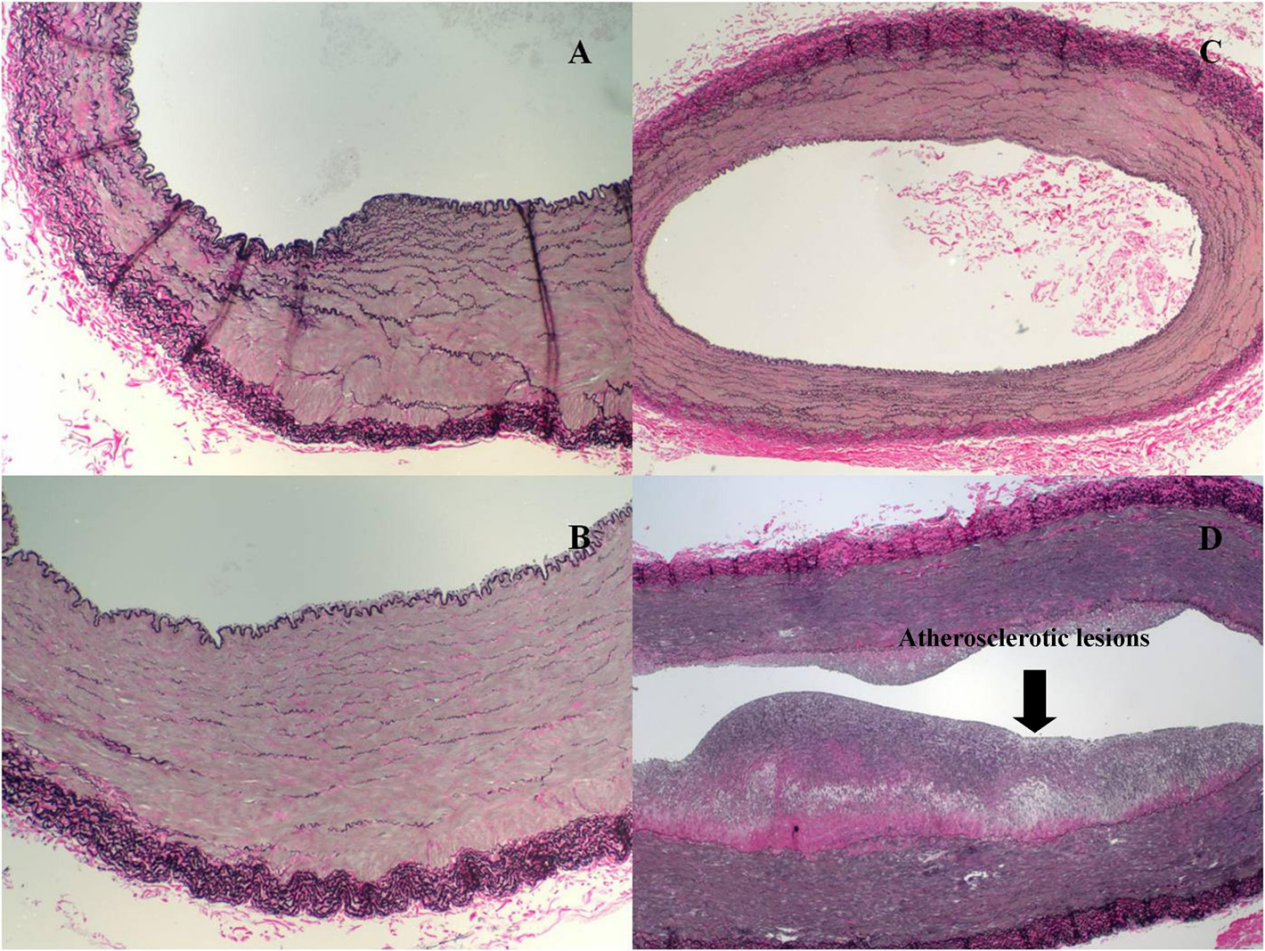
Representative VerhoeffVanGiesson (VVG) stains at 100x (A and B) and 40x (C and D) from brachial and femoral arteries of nine month (A, B) and two year old (C, D) Rapacz FHC swine.

Artery sections were stained with VVG and H/E to quantify the severity of atherosclerotic disease using Stary classification [6] (Table 1). The brachial arteries of the nine month old animals had no classifiable disease while four femoral arteries had mild disease with one showing mineral infiltration (Stary stage 1 and 2). Two of the five brachial arteries of the two year old swine had mild disease with Stary stage 1 and 2, respectively (Figure 2). Four femoral arteries of the two year old animals showed advanced disease (Stary stage 4 and 5) indicated by lymphocytes, transmural infiltration, mineral inclusions and foam cells (Figure 2).

**Table 1:** Histological classification of arteries

**Pig#**	**Age (months)**	**Artery**	**Stary stage**	**% Sudan IV**	**Heatmap cluster**	**Comments**
1	9	brachial	0	0.79	4	No lesion
		femoral	2	2.27	1	Mineral present
2	9	brachial	0	0.00	4	No lesion
		femoral	1	0.00	1	
3	9	brachial	0	0.21	4	No lesion
		femoral	1	1.31	1	
4	9	brachial	0	0.00	4	No lesion
		femoral	1	1.07	1	
5	9	brachial	0	0.62	4	No lesion
		femoral	0	0.00	1	
6	24	brachial	0	2.32	3	No lesion
		femoral	0	4.90	3	
7	24	brachial	0	0.46	3	No lesion
		femoral	2	4.69	3	Intimal thickness to 200 microns
8	24	brachial	0	2.84	3	No lesion
		femoral	5	11.99	2	Transmural inflammation, many lymphocytes, fibrous cap to 600 microns thick, intima to 3 mm thick
9	24	brachial	1	6.56	3	
		femoral	4	38.04	2	Intima to 800 microns, cap to 150 microns thick, mostly foam cells with few lymphocytes
10	24	brachial	2	9.46	2	Intima to 600 microns
		femoral	5	53.70	2	Mineral present, intima 1.7 mm thick, cap 600 microns, media disrupted

### Microarray

**Figure 3:**
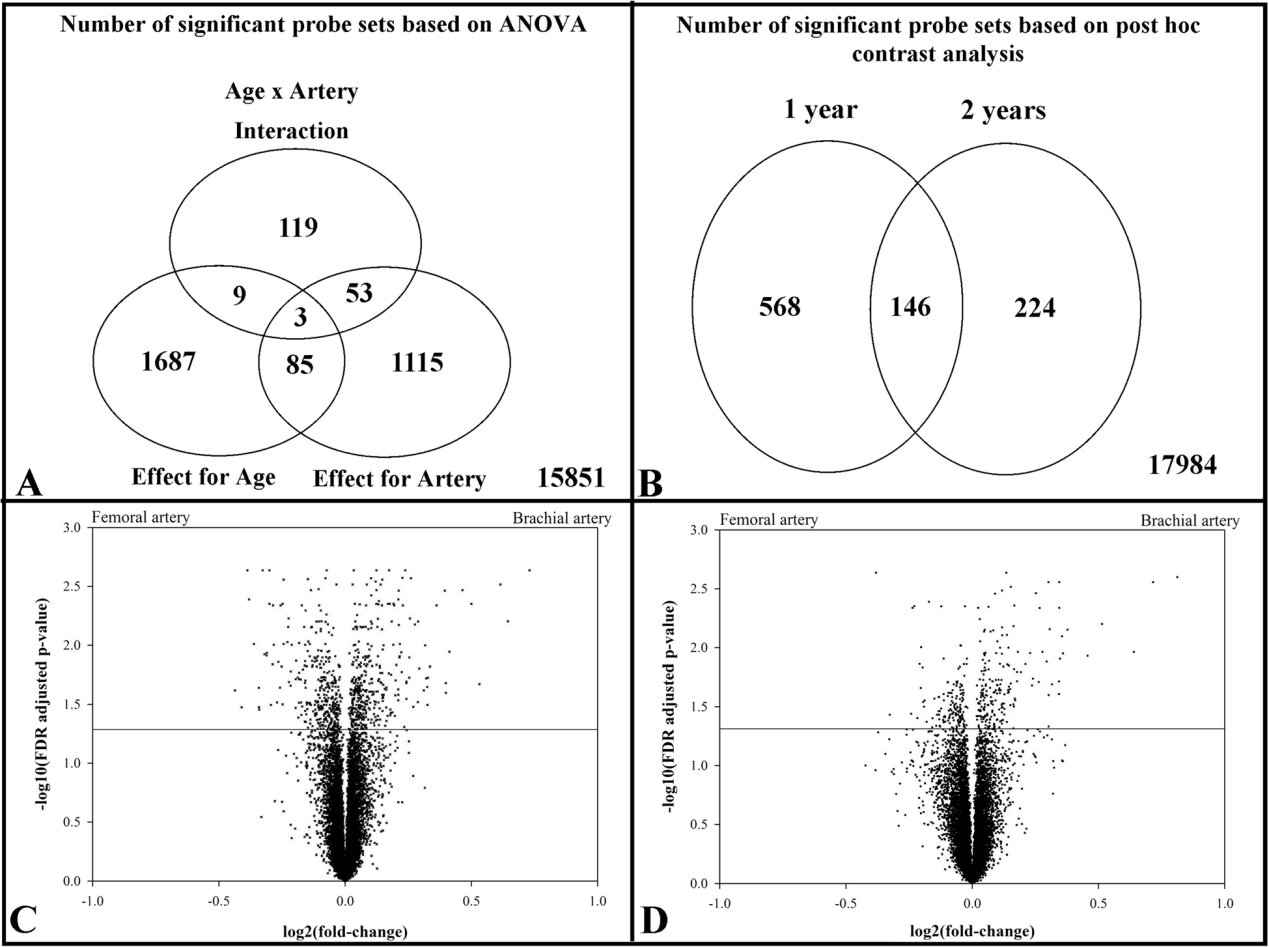
**Microarray results overview.** The Venn diagrams illustrate the results of the 2 x 2 ANOVA **(A)** and the contrast comparison between arteries within age group **(B)**. The volcano plots illustrate the overall microarray data for differential gene expression between brachial and femoral arteries in the nine month **(C)** and two **(D)** year old Rapacz FHC swine. The points indicate the log2(fold-change) (on the x-axis) and the –log10(FDR adj. P value) (on the y-axis) for each probe set with sufficient signal. The horizontal line (y = 1.3) specifies the FDR cut-off for significance (FDR = 0.05).

A total of 18,922 probe sets of the 23,124 on the Affymetrix Porcine Genome Array had sufficient signal for further analysis. A total number of 1784 and 1256 probe sets were identified as having a significant main effect for age and artery respectively, while 184 transcripts had a significant age × artery interaction (Figure 3A) (Additional file 1: Table S1, Additional file 2: Table S2, and Additional file 3: Table S3). A total of 88 genes had a significant effect for age and artery, 12 probe sets had a significant main effect for age and a significant age × artery interaction, 56 transcripts had a significant effect for artery and a significant age × artery interaction, and 3 genes were significant for both main effects and the interaction. The contrast comparison identified 714 and 370 probe sets with differential expression between arteries in one and two year old animals, respectively (Figure 3B). Furthermore, significant differences in transcript abundance for both age groups were observed for 146 genes. Volcano plots show that there is a similar distribution of differentially expressed genes (above the horizontal line, FDR = 0.05) that are down- or up- regulated in the brachial artery relative to the femoral artery in nine month-old pigs (Figure 3C). In the two year old pigs, there is a smaller number of differentially expressed genes with a higher proportion of genes up-regulated in the brachial artery relative to the femoral artery (Figure 3D).

In order to identify genes and pathways which could influence the differential progression of PAD the 56 probe sets with an effect for artery and an age × artery interaction were investigated. Table 2 links the Affymetrix probe set IDs with the human orthologs. A total of 28 functional gene clusters were identified. Selected results of this analysis are given in Table 3. The FDR value in Table 3 is calculated by comparing the total number of genes in a pathway/gene ontology and the number of genes in our set of 56 gene identifiers. Therefore, this is not a test of statistical significance but a qualitative assessment of enhanced biological signaling pathways within our set of genes. The cell component and molecular function classification identified 12 and 2 genes, respectively, to extracellular function. Biological processes involved in the differentially expressed genes included biological adhesion (7 genes), immune response (6 genes) and phosphorylation (3 genes). The gene ontology analysis also recognized four genes to be involved in vascular smooth muscle contraction and three in calcium signaling.

**Table 2:** Annotated probe sets with a significant effect for artery and age x artery interaction

**Probe Set ID**	**Gene Symbol**	**Gene Description**	**Bits**^**1**^	**E-value**^**2**^	**Effect for age FDR**^**3**^	**Effect for artery FDR**^**3**^	**Interaction effect FDR**^**3**^	**1 year contrast FDR**^**3**^	**2 year contrast FDR**^**3**^	**Human ortholog**	**Heatmap cluster**
Ssc.14126.1.A1_at	FAM65B^5^	Homo sapiens family with sequence similarity 65, member B	238	0	0.07	0.01	0.03	0.01	0.55	FAM65B	A
Ssc.15285.1.S1_at	TPPP^5^	Bos taurus tubulin polymerization promoting protein	186	1.00E-44	0.69	0	0.02	0	0.09	TPPP	A
Ssc.31132.1.A1_at	TRAF3IP2^5^	Bos taurus TRAF3 interacting protein 2	1370	0	0.91	0.04	0.03	0.01	0.98	TRAF3IP2	A
Ssc.5052.1.S1_at	FKBP15^5^	Homo sapiens FK506 binding protein 15	2159	0	0.32	0.02	0.04	0.72	0.02	FKBP15	A
Ssc.15541.1.A1_at	PAMR1^5^	Bos taurus regeneration associated muscle protease	1929	0	0.41	0.02	0.04	0.01	0.68	PAMR1	B
Ssc.16614.1.A1_at	PPP1R12A^4^	Protein phosphatase 1 regulatory subunit 12A			0.07	0.01	0.04	0.4	0.01	PPP1R12A	B
Ssc.21536.2.S1_at	PDE5A^6^	Homo sapiens phosphodiesterase 5A, cGMP-specific	282	2.00E-64	0.36	0	0.05	0.03	0	PDE5A	B
Ssc.21845.1.S1_at	OPHN1^4^	Oligophrenin 1			0.91	0.04	0.04	0.98	0.02	OPHN1	B
Ssc.10497.1.S1_at	SCUBE3^6^	signal peptide, CUB domain, EGF-like 3	526	8.00E-146	0.2	0.03	0.04	0.01	0.96	SCUBE3	C
Ssc.15046.1.A1_at	NCOA7^5^	Bos taurus nuclear receptor coactivator 7	498	0	0.12	0.01	0.05	0.01	0.36	NCOA7	C
Ssc.17488.1.S1_at	PAN3^4^	poly(A) specific ribonuclease subunit homolog			0.02	0.01	0.02	0.43	0.01	PAN3	C
Ssc.21735.1.S1_s_at	NPHP1^5^	Bos taurus nephronophthisis 1	615	0	0.31	0.01	0.03	0.66	0.01	NPHP1	C
Ssc.8097.1.A1_at	RAB18^5^	RAB18, member RAS oncogene family	322	2.00E-84	0.1	0.01	0.01	0	0.83	RAB18	C
Ssc.1205.1.S1_at	NR2F2^5^	Macaca mulatta nuclear receptor subfamily 2, group F, member 2, transcript variant 2	438	0	0.34	0.03	0.05	0.01	0.9	NR2F2	D
Ssc.21644.1.S1_at	STT3A^5^	PREDICTED: Pan troglodytes integral membrane protein 1, transcript variant 3	98	9.00E-18	0.44	0.03	0.03	0.01	0.99	SST3A	D
Ssc.9029.1.S1_at	CST3^5^	Homo sapiens cystatin C	153	3.00E-34	0.16	0.05	0.01	0.3	0.01	CST3	D
Ssc.17115.1.A1_at	TMEM43^5^	Bos taurus transmembrane protein 43	1403	0	0.68	0.01	0.03	0	0.39	TMEM43	E
Ssc.19358.1.S1_at	ZDHHC9^5^	Homo sapiens zinc finger, DHHC-type containing 9	1834	0	0.31	0.04	0.05	0.95	0.03	ZDHHC9	E
Ssc.5605.1.A1_at	ITPR1^4^	Inositol 1,4,5-trisphosphate receptor type 1			0.34	0.04	0.02	0.54	0.02	ITPR1	E
Ssc.1362.1.S1_at	DBNL^5^	Bos taurus drebrin-like	785	0	0.29	0.02	0.03	0.91	0.01	DBNL	F
Ssc.16682.1.S1_at	RRP1B^6^	Homo sapiens ribosomal RNA processing 1 homolog B	394	9.00E-95	0.69	0.05	0.05	0.99	0.03	RRP1B	F
Ssc.27980.1.A1_at	FAM70A^5^	Homo sapiens family with sequence similarity 70, member A	579	0	0.07	0.01	0.03	0.37	0.01	FAM70A	F
Ssc.29681.1.A1_at	CCDC48^6^	Bos taurus coiled-coil domain containing 48	68	1.00E-06	0.43	0.02	0.05	0.01	0.65	CCDC48	F
Ssc.3348.1.S1_at	CD97^5^	Homo sapiens CD97 molecule	517	0	0.02	0	0.05	0	0.04	CD97	F
Ssc.4643.1.A1_at	NR4A2^5^	Homo sapiens nuclear receptor subfamily 4, group A, member 2	400	0	0.03	0.02	0.05	0.01	0.77	NR4A2	F
Ssc.5113.2.A1_at	RPE^5^	Bos taurus ribulose-5-phosphate-3-epimerase	204	0	0.41	0	0.04	0.01	0	RPE	F
Ssc.6463.1.A1_at	MYD88^5^	Homo sapiens myeloid differentiation primary response gene	658	0	0.2	0.03	0.04	0.95	0.02	MYD88	F
Ssc.10406.1.A1_at	THBS1^4^	Thrombospondin 1 precursor			0.13	0	0.03	0	0.04	THBS1	G
Ssc.13002.1.S1_at	PPP1R14A^5^	Sus scrofa protein phosphatase 1, regulatory (inhibitor) subunit 14A	521	0	0.61	0.05	0.05	0.99	0.03	PPP1R14A	G
Ssc.1509.1.S1_at	TNFRSF21^6^	Bos taurus tumor necrosis factor receptor superfamily, member 21	1199	0	0.36	0.05	0.04	0.02	0.97	TNFRSF21	G
Ssc.17903.1.A1_at	ACYP1^4^	Acylphosphatase, organ-common type isozyme			0.71	0.01	0.04	0	0.17	ACYP1	G
Ssc.22406.1.A1_at	AQP1^5^	Bos taurus aquaporin 1	1211	0	0.27	0.01	0.05	0.01	0.46	AQP1	G
Ssc.4707.1.A1_at	KITLG^5^	Homo sapiens KIT ligand	161	1.00E-36	0.16	0.03	0.03	0.01	0.99	KITLG	G
Ssc.5844.1.A1_at	PTCD3^5^	Homo sapiens Pentatricopeptide repeat domain 3	1600	0	0.06	0.04	0.02	0.01	0.6	PTCD3	G
Ssc.6788.1.A1_at	SORBS2^6^	Homo sapiens sorbin and SH3 domain containing 2	452	1.00E-123	0.34	0.02	0.04	0.01	0.59	SORBS2	G
Ssc.8683.1.S1_at	PLN^5^	Bos taurus phospholamban	129	5.00E-27	0.17	0	0.03	0.02	0	PLN	G
Ssc.9527.1.S1_at	NPNT^5^	Homo sapiens nephronectin	1017	0	0.28	0.01	0.03	0	0.28	NPNT	G
Ssc.9714.2.S1_at	LMO4^4^	Bos taurus LIM domain only 4	747	0	0.61	0.01	0.05	0	0.19	LMO4	G
Ssc.17370.1.A1_at	ADRA1B^5^	Homo sapiens adrenergic, alpha-1B-, receptor	133	2.00E-28	0.14	0	0.05	0	0.01	ADRA1B	H
Ssc.20476.1.S1_at	CR1L^4^	Complement C4b binding protein CR-1 like			0.41	0.01	0.04	0	0.15	CR1L	H
Ssc.26444.1.A1_at	FARP1^6^	Bos taurus FERM, RhoGEF (ARHGEF) and pleckstrin domain protein 1	533	5.00E-148	0.09	0.01	0.05	0.01	0.34	FARP1	H
Ssc.3034.1.S1_at	SCAMP2^5^	Homo sapiens secretory carrier membrane protein 2	1231	0	0.55	0.02	0.02	0.93	0.01	SCAMP2	H
Ssc.4216.1.S1_at	RNPEP^5^	Homo sapiens arginyl aminopeptidase	1451	0	0.26	0.04	0.04	0.92	0.03	RNPEP	H
Ssc.8492.1.A1_at	RIMS2^4^	Regulating synaptic membrane exocytosis protein 2			0.94	0.05	0.03	0.01	0.84	RIMS2	H
Ssc.13576.1.S1_at	IFT57^5^	Bos taurus intraflagellar transport 57 homolog	1982	0	0.09	0.02	0.02	0.89	0.01	ITF57	I
Ssc.24231.1.S1_at	CBL^5^	Homo sapiens Cas-Br-M (murine) ecotropic retroviral transforming sequence	204	0	0.87	0.02	0.05	0.69	0.02	CBL	I
Ssc.29996.1.A1_at	DPP10^4^	dipeptidylpeptidase 10 isoform1			0.68	0.01	0.02	0.72	0.01	DPP10	I
Ssc.23484.1.A1_a_at	SCARB2^5^	Bos taurus scavenger receptor class B, member 2	141	7.00E-31	0.08	0.05	0.04	0.01	0.93	SCARB2	J
Ssc.24920.2.S1_a_at	DUS2L^5^	Bos taurus dihydrouridine synthase 2-like, SMM1 homolog	1528	0	0.12	0.02	0.04	0.57	0.01	DUS2L	J
Ssc.5587.1.A1_at	NID1^5^	Homo sapiens nidogen 1	262	0	0.86	0	0.01	0.29	0	NID1	J
Ssc.21655.1.A1_at	GDNF^5^	Mus musculus glial cell line derived neurotrophic factor	202	0	0.11	0	0.03	0	0.1	GDNF	K
Ssc.9436.1.A1_at	SETBP1^5^	Homo sapiens SET binding protein 1	232	0	0.06	0	0.02	0	0.14	SETBP1	K
Ssc.10966.1.A1_at	RBBP7^4^	Histone acetyltransferase type B subunit 2			0.54	0	0.03	0.06	0	RBBP7	L
Ssc.23899.1.A1_at	MKX^5^	Homo sapiens mohawk homeobox	400	0	0.77	0	0.02	0	0.13	MKX	L
Ssc.8790.1.A1_at	CCL28^4^	Small inducible cytokine A28 precursor			0.52	0.03	0.03	0.95	0.02	CCL28	L
Ssc.14335.1.A1_at	SH2D4B^4^	SH2 domain containing 4B			0.9	0.01	0.02	0.72	0.01	SH2D4B	M

^1^Bit score provides a measure of the quality of the alignment. ^2^Number of hits one can “expect” to see by chance when searching a database of a particular size ^3^False discovery rate. ^4^Annotation from AffymetrixNetAffx.^5^Annotation from ANEXdb: Animal Expression Database. ^6^Human ortholog from NCBI BLASTn.

**Table 3:** Selected DAVID results of the 56 human orthologs of genes with a significant effect for significantly different levels of transcript abundance between arteries in both age groups

	**Term**	**Description**	**Count**	**FDR**	**Genes**
GO cell component	GO:0005576	extracellular region	12	0.9679	CD97, SCUBE3, NPNT, PAMR1, CST3, KITLG, NID1, THBS1, GDNF, CCL28, RNPEP, CR1L
	GO:0005856	cytoskeleton	9	0.9194	FAM65B, DBNL, SORBS2, TPPP, OPHN1, KITLG, FKBP15, FARP1, ITPR1
GO biological process	GO:0022610	biological adhesion	7	0.8785	CD97, NPNT, KITLG, NID1, SCARB2, THBS1, NPHP1
	GO:0006955	immune response	6	0.9087	CD97, TRAF3IP2, DBNL, MYD88, THBS1, CCL28
	GO:0043405	regulation of MAP kinase activity	3	0.92	DBNL, KITLG, THBS1
GO molecular function	GO:0005509	calcium ion binding	7	0.9999	CD97, SCUBE3, NPNT, CBL, NID1, THBS1, ITPR1
	GO:0008092	cytoskeletal protein binding	5	0.9968	DBNL, SORBS2, TPPP, OPHN1, FARP1
	GO:0050840	extracellular matrix binding	2	0.9915	NID1, THBS1
KEGG pathway	hsa04270	Vascular smooth muscle contraction	4	0.1137	ADRA1B, PPP1R12A, PPP1R14A, ITPR1
	hsa04020	Calcium signaling pathway	3	0.8054	PLN, ADRA1B, ITPR1

**Figure 4:**
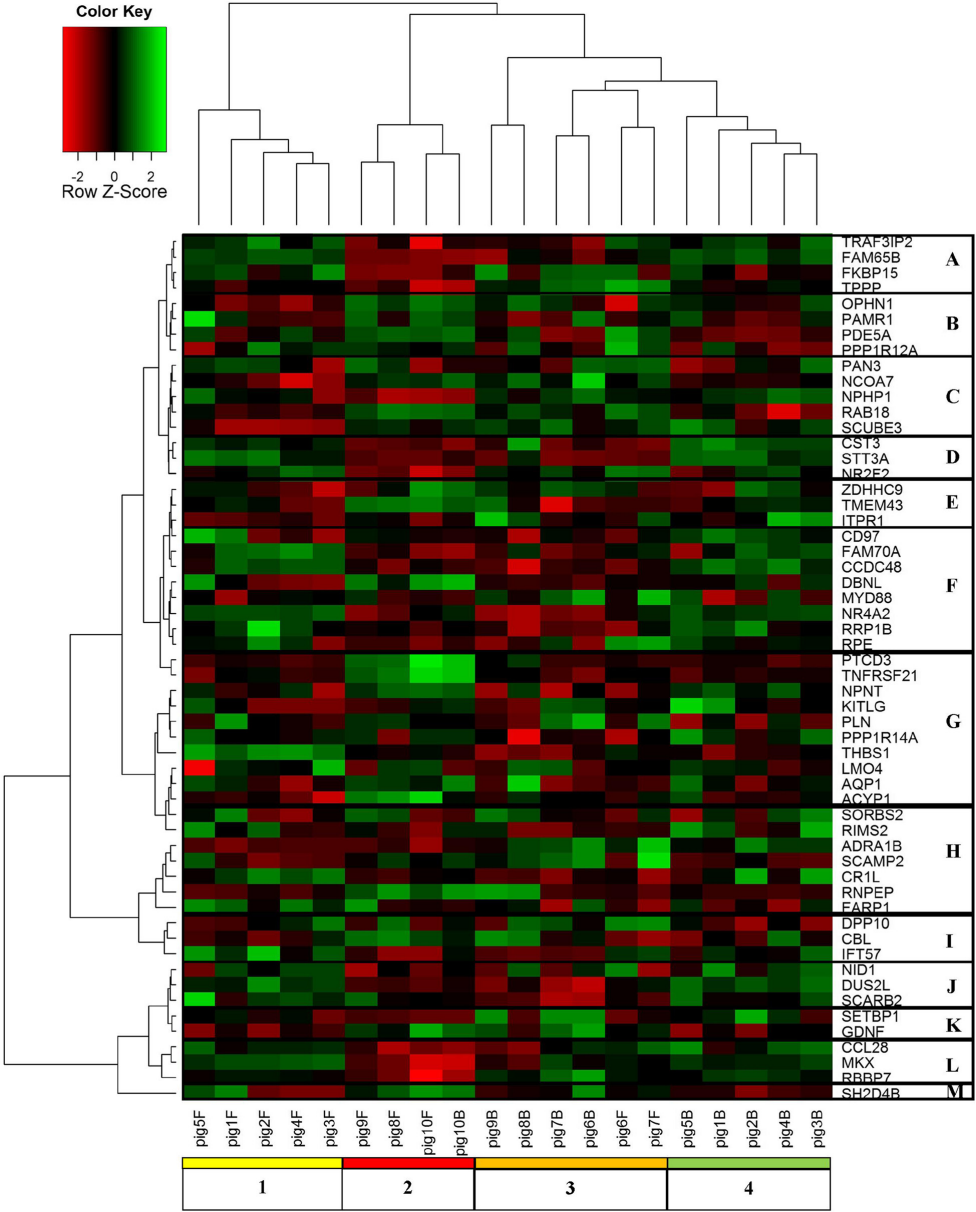
**Heatmap displaying the 56 probe sets with a significant effect for artery and age x artery interaction.** The red and green color of the cells indicates relative transcript abundance below and above the average gene expression for that particular probe set, respectively. The columns represent the 22 samples while the rows correspond to the 56 gene IDs. The dendograms on the *left* and *top* use two-way hierarchical clustering to cluster samples and probe sets, respectively, according to similarity in change in relative transcript abundance. Specifically, a total of four and 13 distinct clusters were identified for the samples (denoted “1”,”2”,”3”, and “4”) and probe sets (denoted “A” through “M”). The description under each column denotes the animal ID from Table 2 and “B” for brachial or “F” for femoral artery.

Two-way hierachicalclustering, visualized in the heatmap, groups probe sets with similar change in gene expression across the brachial and femoral artery samples with similar disease progression (Figure 4). Samples and genes within a cluster show similar gene expression profiles. Four artery disease progression clusters were determined by hierarchical clustering (identified by “heatmap cluster” column in Table 2). Cluster *1* and *4* contain nine month- old femoral and brachial arteries, respectively that were healthy or had mild disease. Cluster *2* contains three femoral arteries with advanced disease and the one brachial artery with Stary stage 2 atherosclerosis from two year old swine. Cluster *3* is comprised of four two year old brachial and two femoral arteries with mild disease (Stary stage 1 through 2).

Genes within a cluster, identified using the left dendogram, are likely to be co-regulated and involved in related biological functions that correspond to disease progression or atherosusceptability (Table 2 links gene ID and cluster location in the heatmap). We identified a total of 13 gene clusters. The green cells in cluster *F* (CD97 molecule [CD97], family with sequence similarity 70, member A [FAM70A], coiled-coil domain containing 48 [CCDC48], drebrin-like [DBNL], myeloid differentiation primary response gene [MYD88], nuclear receptor subfamily 4, group A, member 2[NR4A2], ribosomal RNA processing 1 homolog B [RRP1B], ribulose-5-phosphate-3-epimerase [RPE]) and cluster *J* (nidogen 1[NID1], dihydrouridine synthase 2-like, SMM1 homolog [DUS2L], scavenger receptor class B, member 2 [SCARB2]) had higher transcript abundance in healthy nine month old brachial and femoral arteries (vertical clusters 1 and 4) compared to diseased arteries from two year old swine (vertical clusters 2 and 3). The cells colored red in the horizontal clusters for vertical cluster 2 in Figure 4 show that these genes are also down-regulated with advanced atherosclerotic disease. The genes in cluster *2 B*, (Oligophrenin 1[OPHN1], peptidase domain containing associated with muscle regeneration 1 [PAMR1], phosphodiesterase 5A, cGMP-specific [PDE5A], Protein phosphatase 1 regulatory subunit 12A [PPP1R12A]), and cluster *2 G* (Pentatricopeptide repeat domain 3 [PTCD3], tumor necrosis factor receptor superfamily, member 21 [TNFRSF21], nephronectin [NPNT], KIT ligand [KITLG], phospholamban [PLN], protein phosphatase 1, regulatory (inhibitor) subunit 14A [PPP1R14A], Thrombospondin 1 precursor [THBS1], LIM domain only 4 [LMO4], aquaporin [AQP1], Acylphosphatase [ACYP1], sorbin and SH3 domain containing 2 [SORBS2]), and *I* (dipeptidylpeptidase 10 isoform1 [DPP10], Cas-Br-M (murine) ecotropic retroviral transforming sequence [CBL], intraflagellar transport 57 homolog [IFT57]) were up-regulated with advanced disease. Genes in clusters *2C* and *3C* (poly(A) specific ribonuclease subunit homolog [PAN3], nuclear receptor coactivator 7 [NCOA7], RAB18 member RAS oncogene family [RAB18], signal peptide, CUB domain, EGF-like 3 [SCUBE3]) were predominantly up-regulated with age and disease progression with the exception of nephronophthisis 1 (NPHP1). In addition, two genes (SET binding protein 1 [SETBP1], glial cell line derived neurotrophic factor [GDNF]) in cluster *3 K* were down-regulated during mild atherosclerotic disease in femoral but not brachial arteries. Since co-regulation of biological pathways does not require a uniform directional change of transcript abundance for genes involved in specific biological functions, hierarchical clustering also identifies clusters without uniform changes of transcript levels. For example, clusters *E* (zinc finger, DHHC-type containing 9 [ZDHHC9], transmembrane protein 43 [TMEM43], Inositol 1,4,5-trisphosphate receptor type 1 [ITPR1]), *H* (Regulating synaptic membrane exocytosis protein 2 [RIMS2], adrenergic, alpha-1B-, receptor [ADRA1B], secretory carrier membrane protein 2 [SCAMP2], Complement C4b binding protein CR-1 like [CR1L], arginylaminopeptidase [RNPEP], FERM, RhoGEF (ARHGEF) and pleckstrin domain protein 1 [FARP1]), and *M* (SH2 domain containing 4B [SH2D4B]) do not show a clear directional change, indicating that these genes may be co-regulated without a common directional change in response to atherosclerotic disease.

The microarray data was validated using quantitative PCR on probe sets with a significant effect for artery. Overall 8 out of the 9 tested transcripts had significant differential expression (P < 0.05) between arteries by quantitative PCR (Table 4). Furthermore, iroquoishomeobox 1 (IRX1) also showed an effect for age using both methods. Tumor necrosis factor receptor superfamily, member 21 (TNFRSF21) had an age × artery effect by both microarrays and quantitative PCR. An effect for artery only was observed for bone morphogenetic protein 3 (BMP3), potassium voltage-gated channel, Isk-related family, member 1 (KCNE1), angiotensin II receptor, type 1 (AGTR1), G protein-coupled receptor, family C, group 5, member C (GPRC5C), formin homology 2 domain containing 3 (FHOD3), and proproteinconvertase subtilisin/kexin type 5 (PCSK5) for both methods. One probe set did not show an effect for artery (Ssc.17896.2.A1_at). However, the comparison between microarray data and quantitative PCR given shows a high degree of concurrence indicating the relative measurements of the microarray data do correspond to reliable changes in transcript abundance of the genes measured.

**Table 4:** Comparison of absolute PCR results with relative gene transcript abundance of Affymetrix Porcine Microarray

		**Microarray**			**qPCR**		
**Probe Set ID**	**Gene Identifier**	**Effect for artery**	**Effect for age**	**Age x artery interaction**	**Effect for artery**	**Effect for age**	**Age x artery interaction**
Ssc.17846.1.A1_at	IRX1	<0.01	0.03	0.53	<0.01	<0.01	0.18
Ssc.27181.1.S1_at	BMP3	<0.01	0.07	0.16	<0.01	<0.01	0.70
Ssc.1509.1.S1_at	TNFRSF21	0.04	0.35	0.04	0.01	0.29	<0.01
Ssc.15995.2.S1_at	KCNE1	0.04	0.38	0.72	<0.01	0.09	0.74
Ssc.19059.1.A1_at	AGTR1	<0.01	0.87	0.20	<0.01	0.76	0.06
Ssc.12176.3.S1_at	GPRC5C	<0.01	0.80	0.24	<0.01	0.35	0.18
Ssc.17896.1.A1_at	FHOD3	<0.01	0.12	0.87	<0.01	0.09	0.56
Ssc.10078.A1_at	PCSK5	<0.01	0.22	0.11	<0.01	<0.01	<0.01
Ssc.17896.2.A1_at	FHOD3	<0.01	0.09	0.77	0.19	0.03	0.43
	RPLP0				0.42	0.22	0.41

## Discussion

Atherosclerotic lesions are not evenly distributed throughout the vascular system [3]. For example, in humans atherosclerotic lesion development occurs predominantly in the lower limbs while the upper limbs are protected from advanced disease [3,6]. Location dependent hemodynamic environments and a blood pressure gradient due to upright posture and bipedal locomotion are partially responsible for this heterogeneous disease distribution in humans [3,7-9]. However, in swine a human-like atherosclerotic disease distribution has been observed despite the absence of the blood pressure gradient [3-6,10] and similar mean arterial blood pressure, mean blood velocity, and shear rate [10]. Therefore, hemodynamics cannot fully account for the heterogeneous disease distribution between porcine brachial and femoral arteries. We recently identified 430 differentially expressed (DE) genes associated with biological adhesion and Ca-signaling between these arteries in 13 day old healthy swine [20]. Therefore, we now hypothesized that these differences in healthy arteries would enable rapid disease development in femoral but not brachial arteries. This proposed divergence in atherosclerotic phenotype, classified by Sudan IV, VVG, and H/E staining, between arteries would be accompanied by an increasing number of DE genes with disease progression. In contrast to our hypothesis, the total number of differential transcripts between arteries was reduced with the heterogeneous progression of atherosclerotic disease. However, 56 genes with differential abundance throughout atherosclerotic disease progression are most likely to influence the heterogeneous lesion distribution. In agreement with our previous results [20], this study also reports that these genes are associated with calcium signaling, extracellular function, and biological adhesion.

Previous research has shown that Rapacz FHC swine rapidly develop human-like peripheral atherosclerotic disease due to an allelic variant for apolipoprotein B and a low-density lipoprotein receptor dysfunction [17-19]. Despite similar mean arterial blood pressure, mean blood velocity, and shear rate, this species develops severe atherosclerosis in femoral but not brachial arteries [10]. We assessed atherosclerotic disease distribution using quantitative and qualitative methods. Firstly, Sudan IV staining for lipid depositions in the endothelial and sub-endothelial layer showed no differences between brachial and femoral arteries in nine month-old animals. In two year-old swine a trend (p = 0.09) towards increased lipid depositions in femoral compared to brachial arteries was observed. VVG and H/E staining allow for Stary stage classification [21] and a qualitative assessment of disease progression. The brachial arteries of the nine month-old animals were classified as healthy (Stary stage zero). Femoral arteries from this age group had healthy arteries or very mild disease (Stary stage 1) with the exception of one artery with mineral infiltration (Stary stage two). Brachial arteries from two year-old swine exhibited no (three arteries) or very mild atherosclerotic disease (two arteries). However, the rapid atherosclerotic disease progression in the femoral artery was seen in three of the five two year-old animals. Overall, quantitative and qualitative histological atherosclerotic disease classification using Sudan IV and Stary classification verified that brachial arteries are protected from advanced disease, while femoral arteries rapidly develop atherosclerotic lesions in Rapacz FHC swine. Therefore, the arterial phenotype provided a large spectrum of disease states (healthy, mild and advanced atherosclerosis) for gene expression profiling.

We hypothesized that the number of differential transcripts between arteries would increase due to the rapid disease progression in femoral but not brachial arteries. Contrary to this hypothesis, our current investigation demonstrates that the number of DE genes between arteries decreases with a diverging disease progression. With substantial differences in atherosclerotic disease between arteries of two year old swine, 370 DE genes were identified. This is a reduction of 344 probe sets compared to the number of DE genes for nine month-old animals (Figure 2B). Although 17,984 detectable transcripts did not change with age and disease progression in this study, a reduction of more than 50% of the total number of DE transcripts within the same tissue type is significant. Our findings are supported by a previous cross-sectional study which investigated gene expression patterns of human ECs from early and advanced atherosclerosis compared to healthy controls [22]. Specifically, this study reported a total of 713 DE genes between ECs from mild atherosclerotic disease compared to those from healthy controls. However, when ECs from advanced atherosclerosis were compared to healthy controls, only 403 transcripts reached significantly different levels of relative transcript abundance. Therefore, similar to our findings in whole arteries, the number of DE transcripts in ECs decreased despite a diverging disease classification. This is remarkable since the development of atherosclerosis is generally accompanied by an up-regulation of inflammatory genes in the diseased arteries [22]. In addition to the reduced number of transcripts, the volcano plot for the two year old animals shows a larger number of up-regulated genes in the healthier brachial arteries rather than the diseased femoral arteries. Overall, Volgers et al. [22] and our findings suggest that differences in disease phenotype do not have to be accompanied by an increased number of DE genes.

Quantitative differences in total number of DE transcripts between samples do not compare gene expression signatures within arteries with a variety of disease states. For this purpose we analyzed the 56 genes with a significant main effect for age as well as a significant age × artery interaction using two-way hierarchical clustering. The heatmap, visualizing the clustering results, groups arteries from nine month old animals (cluster *1* and *4*) furthest away from each other, even though brachial arteries in this age group are healthy and femoral arteries only show very mild signs of disease (i.e. low Stary stage and Sudan IV staining). This is in agreement with our previous study reporting 430 DE probe sets between healthy brachial and femoral arteries in 13 day old swine [20]. Gene expression profiling for ECs from atheroprotective (iliac) and –susceptible (coronary) arteries in the central circulation reported 51 genes with significantly different levels of transcript abundance [16]. Furthermore, some genes persisted to have differential expression after cell culture [23]. The authors concluded that these differences are due to embryonic vascular bed origin. Even though current knowledge about peripheral artery development is sparse, based on ours and previous results, one may speculate that developmental differences during angio- and vasculogenesis contribute to our observed pattern of differential gene expression between brachial and femoral arteries in Rapacz FHC swine.

Additional support for this speculation can be derived from arteries of the two year-old swine which are grouped close together in clusters *2* and *3*, despite brachial arteries being healthy or having mild disease and femoral arteries showing severe atherosclerotic lesions. Specifically, brachial arteries were characterized with very low Stary stages (0 to 2), which are generally considered reversible, while femoral arteries had lymphocyte infiltration, calcification, and fibrous caps. Considering the large number of DE genes in healthy arteries from young animals, the divergent disease pattern despite a converging gene expression signature in two year old animals and the reported persistent number of DE genes from cultured ECs of porcine iliac and coronary arteries, allows for the speculation of an artery specific developmentally programmed atherosusceptibility. This is also supported by aortic homograft transplantation experiments in which segments from atheroresistant parts of the aorta were exchanged for atherosusceptible segments [14]. The atherosusceptible segments developed severe atherosclerosis even when exposed to an atheroprotective hemodynamic environment while the atheroprotective segments did not develop the disease even when placed in previously identified atherosusceptible regions of the aorta [14]. Therefore, the underlying genetic predisposition determined atherosusceptibility rather than the hemodynamic environment [14]. The current findings of a larger number of DE genes in nine month- compared to two year-old animals and the previous identification of 430 DE genes in healthy 13 day old swine [20] supports the hypothesis of developmentally programmed atherosusceptibility.

Investigating gene networks in atherosclerosis provide a tool to explore the differential disease progression between brachial and femoral arteries. Hierarchical clustering groups genes according to similarity in abundance to identify enriched biological signaling pathways which may contribute to the heterogeneous progression of PAD. We identified a total of 13 gene expression clusters using the left dendogram (Figure 4). Five clusters (*A*, *D*, *F*, *J*, and *L*) are down-regulated, while three clusters (*B*, *G*, *M*) are up-regulated with severe disease (horizontal cluster *2*). However, in addition to being down-regulated in arteries with very advanced disease, clusters *F* and *J* are also down-regulated in two year old arteries with mild disease (red color in vertical cluster *3*). Therefore, whether the expression levels of these genes changed in response to disease or age cannot be determined. Nonetheless, these clusters contain five down- (TRAF3IP2, CST3, CD97, DBNL, MYD88, and CCL28) and one up-regulated gene (THBS1) which are associated with immune response, while biological adhesion was enhanced with CD97 and NID1 being down- and NPNT, KITLG, and THBS1 being up-regulated. This is consistent with previous reports about an involvement of inflammatory genes in atherosclerotic porcine coronary arteries [15] as well as atherosclerotic disease progression in mice and humans [24]. Interestingly, our data also indicates alterations in vascular smooth muscle contraction (up-regulation of PPP1R12A and PPP1R14A) in arteries with very advanced atherosclerotic disease. This demonstrates that in addition to previously identified biological signaling pathways, which play a role in atherosclerotic disease progression, differences in vascular smooth muscle contraction, mediated by alterations in calcium signaling, may also be related to the heterogeneous progression of PAD.

Gene expression differences between healthy and atherosclerotic arteries are important to understand cellular processes involved in the progression and development of atherosclerotic disease. However, the comparison of transcript abundance in nine month-old animals, when atherosclerotic disease differences are minimal, may identify genes predisposing arteries to rapid disease development. Our results identify cluster *C* (PAN3, NCOA7, NPHP1, RAB18, SCUBE3) and three genes (PPP1R14A, THBS1, SH2D4F) to be up-regulated in femoral as well as DBNL and ITPR1 up-regulated in brachial arteries of nine month-old swine, respectively. We previously identified SCUBE3 to have differential abundance in healthy arteries from 13 day old swine [20]. Furthermore, THBS1 is known to influence atherosusceptibility. THBS1 is an antagonist of nitric oxide (NO) signaling [25]. Recent evidence of cultured human vascular smooth muscle cells identified THBS1 as an important pro-atherosclerotic factor that augments atherosclerotic disease progression [26]. Hence, THBS1 may sufficiently decrease NO-signaling in femoral arteries and thereby increase its susceptibility to atherosclerotic disease. However, molecular pathways during the early stages of atherosclerosis are not very well understood. This is exemplified by a functional comparison of porcine brachial and femoral artery endothelium-dependent and –independent vasorelaxation that reported high functional correlation between arteries, but no correlation in a variety of pro- and anti-atherogenic proteins [27]. Even though, our descriptive study does not allow for a definite identification of potentially atheroprotective or -susceptible genes, we demonstrate that there are substantial differences in gene expression between brachial and femoral arteries in 13 day [20] and nine month-old animals prior to the onset of disease which could affect arterial atherosusceptibility later in life.

A limitation of the current investigation is that we did not measure the hemodynamic stimuli in this set of nine month- and two-year old Rapacz FHC swine. However, previous studies have reported a similar mean arterial blood pressure, mean blood velocity, and shear rate in brachial and femoral arteries of Yucatan miniature swine [10]. Even though Rapacz FHC swine are larger compared to Yucatan swine, we assume that the observed hemodynamic similarities between arteries are not affected by size since local artery geometry is unlikely to vary within a species. Nonetheless, our assumption needs to be verified by future research. Our approach of using whole arterial samples implies that a variety of different cell types throughout the progression of atherosclerotic disease may be responsible for our observations. Thus, we cannot determine which type of cell is responsible for the reported differential gene expression. Furthermore, we are aware of the fact that a limitation of this study is the lack of protein data possibly identifying posttranscriptional factors influencing artery specific disease progression. Lastly, an inherent limitation of DAVID analyses is their bias towards previously identified biological signaling pathways.

## Conclusion

The heterogeneous progression of PAD is currently not very well understood. Even though, a blood pressure gradient and unique local hemodynamic environments influence disease susceptibility in humans, swine have a human-like disease distribution in the absence of these factors. This study employs quantitative and qualitative histological methods to confirm an atheroprotected brachial and –susceptible femoral artery phenotype in Rapacz FHC swine. We also report that diverging differences in disease classification do not result in an increased number of DE genes. Furthermore, our results indicate that developmental differences between arteries may predispose the femoral artery to rapid disease development. Our results also suggest that differences in vascular smooth muscle contraction influence the differential progression of PAD. Nonetheless, a dysregulation of NO-signaling due to THBS1 may also contribute. Future studies will need to assess how these pathways and genes influence the initiation and progression of atherosclerosis in the peripheral arteries at a molecular level.

## Methods

### Tissue harvest and storage

The tissues used in this study were acquired from the Cardiovascular Research Foundation (nine month old animals) and the University of Missouri (two year old animals). All experiments conducted in animals complied with the “Animal Research: Reporting In Vivo Experiments” (ARRIVE) guidelines. All animals were sexually mature. The sacrifice weight for the nine month and two year old animals was 44.2 (±3.24) kg and 147.4 (±6.02) kg, respectively. Straight sections of the brachial (from axillary artery to bifurcation of radial and ulnar artery) and femoral (distal to the bifurcation of the iliac into the deep and common femoral artery) arteries of six nine month and five two year old Rapacz FHC swine were harvested and flushed with Krebs dissecting solution. The left brachial and femoral arteries were harvested for gene expression analysis and placed in RNAlater (Ambion Inc., Austin, TX, USA) at a 1:5 solution/tissue ratio to inhibit RNA degradation. The femoral arteries of a single nine month old animal could not be used for histology. Therefore, five right brachial and femoral arteries of both age groups were used for histology and fixed in 10 ml of 10% Formalin solution immediately after tissue harvest.

### Histology

A two millimeter section of the right brachial and femoral arteries were cut from the proximal end of each artery and placed in 100% Ethanol for 24 h. Subsequently, the section was processed through Paraffin embedment and sliced at five microns. The remainder of the arteries was returned to the 10% Formalin solution. The sectioned samples were stained with Verhoeff Van-Giesson (VVG) for elastin and Hematoxylin/Eosin (H/E) for collagen. All stains were performed at the Purdue University Histopathology Service Laboratory according to standard protocol. Brachial and femoral artery sections were stained with VVG and H/E to assess atherosclerotic lesion formation by a certified pathologist using the Stary stage classification system [21]. Sudan IV, which stains for lipids on the endothelial and sub-endothelial space of the arteries, was also performed to determine the prevalence of atherosclerosis in brachial and femoral arteries. Briefly, the vessels were cleaned, cut longitudinal, pinned out and washed in Sudan IV solution for 15 min followed by 5 min of decolorization with 70% Ethanol and flushed with distilled water for an hour. Digital photos of the stained and pinned out arteries were acquired and analyzed using Image Pro to determine the percentage of stained vessel surface area (Mediacybernetics, Bethesda, MD, USA). Sudan IV stained percentage surface areas of the nine month and two year old animals were subjected to a paired t-test using SAS9.2 (SAS Institute, Cary, NC, USA) with a significant p-value of α = 0.05.

### RNA Isolation

Whole arteries were homogenized in 4 M guanidinium thiocyanate, 25 mM sodium citrate, 50 mM EDTA, and 1% sodium-N-lauroyl-sarcosine. Total RNA was isolated by ultracentrifugation of the homogenate on a cushion of 5.7 M cesium chloride and 50 mM EDTA [28] followed by further purification and DNase I treatment using NucleoSpin RNA II (Machery Nagel, Dueren, Germany) columns according to the manufacturer’s specifications. After extraction all purified RNA samples were stored in RNA Storage Solution® (Ambion Inc., Austin, TX, USA) at −80°C. Spectophotometry and electrophoresis on an Agilent 2100 Bioanalyzer were used to determine RNA quantity and quality. All samples reached the minimum amount and quality (RIN score > 5.3) of purified total RNA.

### Microarrays

Total purified RNA was hybridized to Affymetrix Porcine Expression Arrays. BiotinylatedcRNA was synthesized from one microgram of purified artery RNA supplemented with GeneChip poly-A factors using the manufacturer’s reagents and protocols (Affymetrix Inc., Santa Clara, CA, USA). Each microarray was hybridized with 20 μg of fragmented cRNA followed by incubation for 16 h at 45˚C with eukaryotic hybridization controls and herring sperm DNA. GeneChips were stained with streptavidin phycoerytherin. The raw images were scanned using the GeneChip Scanner 3000. GeneChip Operating Software (GCOS, Affymetrix Inc., Santa Clara, CA, USA) was used to read and digitally store the image. Microarray data (GSE21043) were deposited in the National Center for Biotechnology Information Gene Expression Omnibus (Bethesda, MD, USA) in accordance with MIAME compliance.

### Microarray Data Analysis

The raw signal intensities were deciphered into CEL-files using the Affymetrix DataExchangeConsole (Affymetrix Inc., Santa Clara, CA, USA). The MAS5 function in Bioconductor was used to determine signal intensity and make present or absent calls for each probe set [29]. Probe sets without detectable signal (absent calls) were excluded from further analysis. The robust multichip average (RMA) function in Bioconductor was used for background correction, normalization, and summarization of the probe level data to generate signal intensities for the present probe sets [30]. Bayesian correction was used on the RMA data to improve signal quality. Transcripts with significant effects for age, artery, and age × artery interaction were identified using a 2 × 2 ANOVA with proc MIXED in SAS 9.2 (SAS Institute, Cary, NC, USA). The contrast option in proc MIXED was used post hoc to identify transcripts with differential gene expression between arteries within age group. Statistical significance was determined by a false discovery rate (FDR) < 0.05 according to Benjamini and Hochberg [31].

Probe sets with a significant effect for artery and an age x artery interaction are likely to influence the differential progression of PAD. Therefore, these transcripts were further analyzed in the AffymetrixNetAffx Analysis Center and assigned to their respective transcripts or genes. Gene Ontology analysis was performed to identify biological signaling pathways enriched in these transcripts using the Database for Annotation, Visualization, and Integrated Discovery (DAVID) [32]. The low rate of annotation of the Affymetrix Porcine Genome Array made the gene ontology analysis of the porcine genes inefficient. Therefore, the human orthologs of transcripts with significant differential expression were used as input identifiers for functional annotation clustering in DAVID. The human orthologs were identified using ANEXdb: Animal Expression Database [33], basic local alignment search tool (BLASTn) with a cut-off value of 1e^-5^, or a published annotation of the Affymetrix Porcine Genome Array [34].

### Quantitative RT-PCR

Primer 3 was used to design primers for quantitative PCR from the most representative public ID sequence specified by Affymetrix annotation [35]. Primer sequences and quantitative PCR conditions are given in Table 5. Complimentary DNA (cDNA) was synthesized from DNase-treated total RNA using random hexamer primers and MMLV reverse transcriptase (Invitrogen, Carlsbad, CA, USA). Primer pairs for quantitative PCR analysis were tested on four brachial and four femoral artery cDNAs using iQ SYBR Green Supermix reagents on an iCycler Real-Time PCR Detection System (Bio-Rad Inc., Hercules, CA, USA). Primer specificity and capture temperature were determined by melt curve analysis. PCR products were cloned into a pCR-4TOPO vector and transformed into TOP10 *E. coli* (Invitrogen, Inc., Carlsbad, CA, USA). Plasmids were sequenced to confirm the identity of each amplicon. Plasmids with sequence-verified inserts were quantified by fluorometry (Picogreen® dsDNA Quantitation Kit, Invitrogen Inc., Carlsbad, CA, USA) for use as quantification standards.

**Table 5:** Quantitative PCR primers and conditions

** Probe Set ID**	** Primer Sequence**	**Annealing Temp.(C)**	**Capturing Temp. (C)**	**Probe Size**	**Gene Identifier**	**Identity%**
Ssc.15995.2.S1_at	GGCATCATGCTGAGTTACATC	58	82	106	KCNE1	100
	AGTAAGCCTTGTCCTTCTCCTG					
Ssc.10078.1.A1_at	CCTGAGCTGAGAGCCTAGATATG	58	79	113	PCSK5	99
	TTGAATGGCTCCTCTTCAGG					
Ssc.1509.1.S1_at	GTTCCAGAACAGCTCGATCC	58	82	155	TNFRSF21	98
	TGCATTGATTCCTTGTGCTC					
Ssc.17846.1.A1_at	ACGACACAAGCACGTGAGG	58	85	157	IRX1	100
	GGCAACGAAGTCAAGGACTC					
Ssc.17896.1.A1_at	TCTCCTCCTTCTGCATCAGC	58	81	126	FHOD3	100
	GCACATGGTCCGTTCTTCTC					
Ssc.17896.2.S1_at	GGCAAGTTCTCTGGCAGTTC	58	84	100	FHOD3	98
	TCAGCACAGCCTTCATGTTC					
Ssc.19059.1.A1_at	TGCAGTAGCAGGTACAATGGAG	58	78	123	AGTR1	99
	ACCTTGAGAGGAGCAACAGG					
Ssc.12176.3.S1_at	AGAGTTCTTGCCTTCCTTCG	58	88	142	GPRC5C	99
	AGGAGCTTACGACGTCATCC					

Quantitative PCR assays were carried out in 15 μL reaction volumes of iQ SYBR Green Supermix with diluted first-strand cDNA equivalent to 100 ng of input RNA. All cDNA samples were assayed in duplicate. Quantification standards were comprised of four 100-fold dilutions of EcoRI digested plasmid DNA (10^7^ to 10^1^ molecules) and were assayed in triplicate. Standards were used to calculate a linear regression model for threshold cycle relative to transcript abundance in each sample. Averaged log values for transcript abundance from sample duplicates were subjected to a 2×2 ANOVA with a significant p-value of α = 0.05 in SAS9.2.

## Competing interests

No conflicts of interest, financial or otherwise, are declared by the authors.

## Authors’ contributions

M.B. – contribution to conception, and design of the experiment; data acquisition, analysis, and interpretation; drafted the manuscript; gave final approval of the manuscript. C.A.B. – data acquisition, analysis, and interpretation; critical revision of the manuscript for important intellectual content; gave final approval of the manuscript. J.H. – data analysis; critical revision of the manuscript for important intellectual content; gave final approval of the manuscript. A.T., G.F.K., J.F.G., C.G.K., J.G.R. – data acquisition; critical revision of the manuscript for important intellectual content; gave final approval of the manuscript. H.M.L. – contribution to conception and design of the experiment; critical revision of the manuscript for important intellectual content; gave final approval of the manuscript. W.v.G.A. – data acquisition, analysis, and interpretation; critical revision of the manuscript for important intellectual content; gave final approval of the manuscript. S.C.N. – conception and design of the experiment; data acquisition, analysis, and interpretation; critical revision of the manuscript; gave final approval of the manuscript. All authors read and approved the final manuscript.

## Supplementary Material

Additional file 1: Table S1 Probe sets with significant effect for age. Click here for file

Additional file 2: Table S2 Probe sets with significant effect for artery. Click here for file

Additional file 3: Table S3 Probe sets with significant effect for age x artery interaction. Click here for file
